# Particle release from implantoplasty of dental implants and impact on cells

**DOI:** 10.1186/s40729-020-00247-1

**Published:** 2020-09-12

**Authors:** Fadi N. Barrak, Siwei Li, Albert M. Muntane, Julian R. Jones

**Affiliations:** 1 Department of Materials, Imperial College London, South Kensington Campus, London, SW7 2AZ UK; 2 School of Dentistry, University of Central Lancashire, Preston, PR1 2HE UK

**Keywords:** Gingival, Fibroblast, Dental implants, Peri-implantitis, Implantoplasty, Titanium, Titanium alloy, Vanadium, Toxicity

## Abstract

**Background:**

With increasing numbers of dental implants placed annually, complications such as peri-implantitis and the subsequent periprosthetic osteolysis are becoming a major concern. Implantoplasty, a commonly used treatment of peri-implantitis, aims to remove plaque from exposed implants and reduce future microbial adhesion and colonisation by mechanically modifying the implant surface topography, delaying re-infection/colonisation of the site. This in vitro study aims to investigate the release of particles from dental implants and their effects on human gingival fibroblasts (HGFs), following an in vitro mock implantoplasty procedure with a diamond burr.

**Materials and methods:**

Commercially available implants made from grade 4 (commercially pure, CP) titanium (G4) and grade 5 Ti-6Al-4 V titanium (G5) alloy implants were investigated. Implant particle compositions were quantified by inductively coupled plasma optical emission spectrometer (ICP-OES) following acid digestion. HGFs were cultured in presence of implant particles, and viability was determined using a metabolic activity assay.

**Results:**

Microparticles and nanoparticles were released from both G4 and G5 implants following the mock implantoplasty procedure. A small amount of vanadium ions were released from G5 particles following immersion in both simulated body fluid and cell culture medium, resulting in significantly reduced viability of HGFs after 10 days of culture.

**Conclusion:**

There is a need for careful evaluation of the materials used in dental implants and the potential risks of the individual constituents of any alloy. The potential cytotoxicity of G5 titanium alloy particles should be considered when choosing a device for dental implants. Additionally, regardless of implant material, the implantoplasty procedure can release nanometre-sized particles, the full systemic effect of which is not fully understood. As such, authors do not recommend implantoplasty for the treatment of peri-implantitis.

## Background

Dental implants offer a viable long-term treatment option for patients with missing teeth [1, 2]. The use of metallic dental implants has relatively high reliability and long-term success rates; however, it is not without complications and the need for ongoing maintenance persists. Particles are generated during the life span of an implant, and this can have significant physiological implications such as disrupted osseointegration and bone resorption (osteolysis) that may in turn lead to implant loss [3, 4]. Particles can be released during implant bed preparation, from implant surface due to shear forces during fixture insertion, from implant-abutment interface due to wear and during functional loading [5, 6]. Exposure to the oral environment such as saliva, bacteria and chemicals such as fluoride can further facilitate the corrosion and degradation of titanium [7–9]. A wide range of 10 to 20 μm in sizes of the released particles were reported in several locations such as at implant surface and peri-implant bone as well as distant sites such as the lungs, liver and kidney [5].

One common issue that can have detrimental impact on the long-term outcome of implant restorations and cause implant failure is peri-implantitis [10]. Peri-implantitis is a plaque-associated pathological condition occurring in tissues around dental implants [11]. It is characterised by inflammation in the peri-implant mucosa and subsequent progressive loss of surrounding supporting bone in which the implant is anchored. The risk of peri-implantitis is dependent on several of factors, and the frequency of peri-implantitis diagnosis has been reported to be 1–47% with selected implant systems [12, 13]. The large variation is due to the criteria used in diagnosis of peri-implantitis, e.g. some diagnose peri-implantitis with 0.5 mm crestal bone loss while at the other extreme 4 mm bone loss is needed for the diagnosis. Moderate to severe peri-implantitis (signs include bleeding on probing/suppuration and bone loss greater than 2 mm) was reported in 14.5% of patients [14]. Recently, the 2017 World Workshop Consensus report stated that in the absence of previous examination that records a diagnosis of peri-implantitis can be made with probing depths of greater than 6 mm and crestal bone loss greater than 3 mm in the presence of bleeding and/or suppuration on gentle probing. However, if previous records are available, then the diagnosis can be made with any increase in pocket depth with post remodelling bone loss of greater than 0.5 mm in the presence of bleeding and/or suppuration on gentle probing [11], A number of studies suggested this inflammatory disease is associated with anaerobic plaque bacteria [15, 16]. It has also been suggested peri-implantitis can also be related to inadequate distribution of the chewing pressure on the tissues surrounding the implant, leading to the loosening of the artificial supports [17]. While plaque is the main risk factor, patients who have history of periodontal disease prior to implant treatment, and those with other risk factors such as poor oral hygiene, smoking or uncontrolled diabetes, also experienced higher rates of peri-implant disease [18, 19]. Additionally, there are also suggestions that peri-implantitis is the result of foreign body reaction [20].

Common treatment options for peri-implantitis include mechanical debridement (with or without antiseptic application) administration of local and systemic antibiotics or surgical techniques. It is well accepted that altering the affected implant surface is necessary to minimise the establishment of biofilm. Implantoplasty involves mechanically modifying the implanted threads (and the rough surface) that have become exposed in the patient’s mouth, due to bone resorption, by removing the outer surface of the metal with rotary instruments, in situ. The purpose is to reduce the roughness of the surface, and it is a commonly used technique to reduce plaque retention and prevent re-infection of the site [21, 22].

To the best of our knowledge, the release of particles from implants following implantoplasty procedure and their effect on cells has not been investigated. The purpose of this in vitro study was to assess the size, composition, ionic product release and biological impact of particles released from commonly used commercially pure grade 4 titanium and grade 5 titanium alloy implants following an implantoplasty procedure with diamond burr. This aims to raise the awareness of potential detrimental side effects of implantoplasty and the need for careful consideration of dental implant material.

## Materials and methods

### Materials

**Fig. 1:**
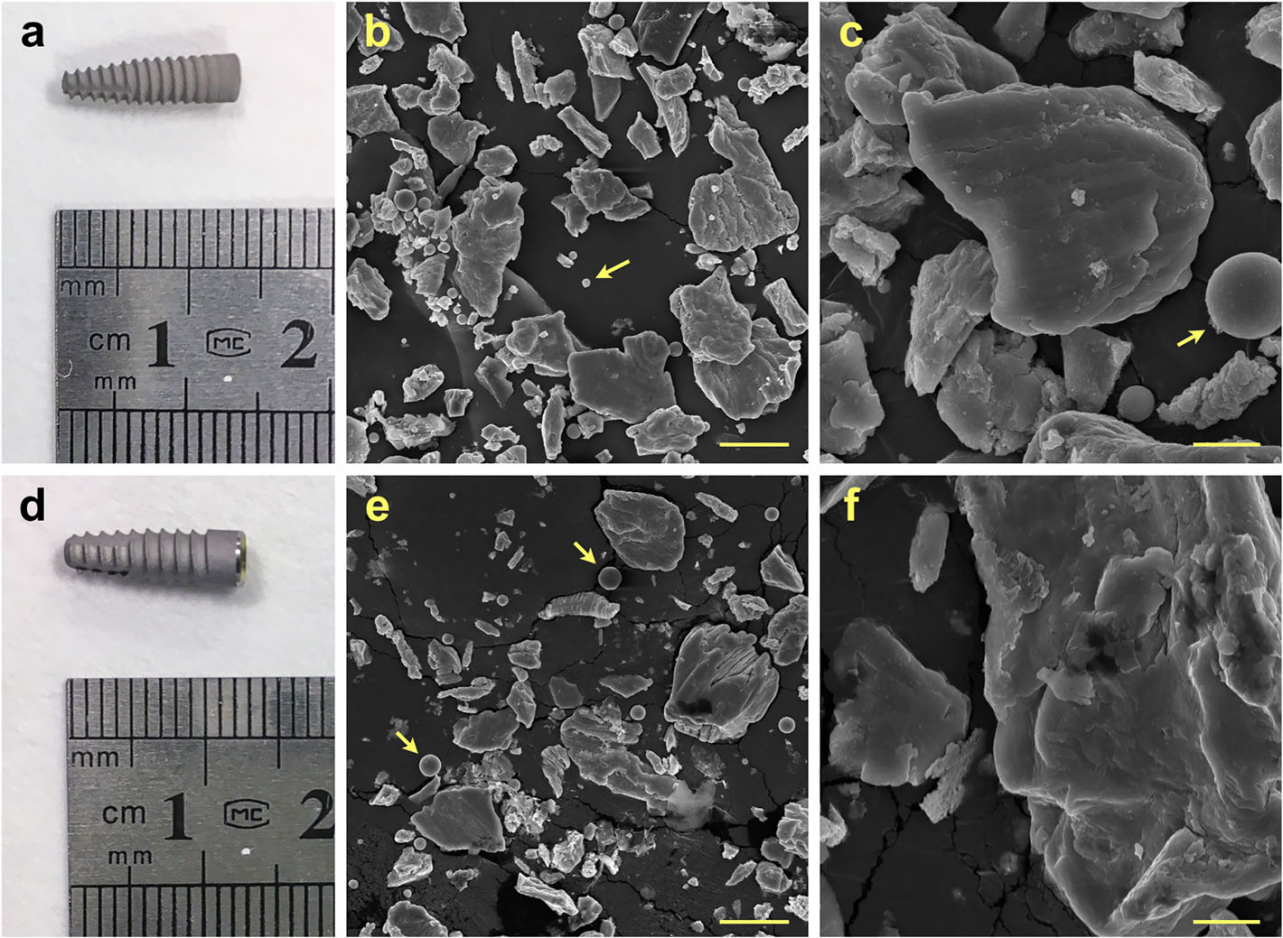
Representative photo of implants and SEM images of particles produced by mock implantoplasty procedure. **a**–**c** Straumann 021.4512, bone level, diameter 4.1 mm, regular CrossFit®, SLA® 12 mm Roxolid® (commercially pure grade 4 titanium). **d**–**f** Biohorizons PBR 50105, RBT 5.0 × 10.5 mm, 5.7 Platform (grade 5 titanium alloy). Arrows indicate titanium oxide spheres. Scale bar represents 20 and 5 μm for low and high magnification respectively

Reagents and solvents were purchased from Sigma-Aldrich (Dorset UK). Commercially pure grade 4 titanium implants (*n* = 3) were purchased from Straumann (Sussex UK, Model number 021.4512, bone-level implant diameter 4.1 mm, Regular CrossFit®, SLA® 12 mm Roxolid®) (Fig. 1a). Grade 5 Ti-6Al-4 V titanium alloy implants were purchased from Biohorizons (Berkshire UK, *n* = 3, model number PBR50105, RBT 5.0 × 10.5 mm, 5.7 Platform) (Fig. 1d).

The methods in this study follow SPQR (standards for reporting qualitative research) guidelines.

### Mock implantoplasty protocol

Implants were secured with forceps held using table clamps. In order to minimise variations in the pressure applied during the implantoplasty, all procedures were completed by one operator. A handpiece (W&H, Alegra dental turbine handpieces TE-98 Led G) was used with motor set at 50,000 rpm. Diamond burrs (Diatech G856.314.021.9ML-200453AA) were limited to single use for 5 min on each implant. Particles released from implants following implantoplasty were collected, weighed and analysed for size, composition and ion release characteristics.

### Dynamic light scattering

Dynamic light scattering (DLS) was performed using a Malvern Zetasizer (instrument 2000) instrument to determine the size distribution of released implant particles. The instrument is equipped with a HeNe laser (*λ* = 632.8 nm) with a backscattering detection angle of 173°. Particle size was also measured using Malvern Mastersizer (Malvern Panalytical Ltd, Royston, UK). Measurement duration (15 min) and number of sub-runs (3) were automatically adjusted by the instrument. Particles were suspended in 70% ethanol at a concentration of 5 mg ml^−1^ and sonicated for 15 min prior to each measurement.

### Scanning electron microscopy

Particle samples were dried in a 60 °C oven and secured to an aluminium sample holder with carbon tape and coated with 10 nm gold. Images were acquired using Zeiss Sigma-300 scanning electron microscope (SEM). For energy dispersive x-ray spectroscopy (EDX) analysis, samples were secured with silver conductive paint.

### Ion release from titanium particles

Simulated body fluid (SBF) was chosen as the dissolution test solution as we were interested in what happens when the particles become embedded in the soft/hard tissue rather than their interaction with saliva. SBF was prepared using the Kokubo method [23, 24]. Seven hundred millilitres of deionised (DI) water in a 1-L polypropylene beaker was warmed to 37 °C in a water bath. The reagents were slowly added to the DI water in the order given in [25], while the solution was continuously stirred. The pH was continuously monitored to avoid precipitation due to sudden increase. Once the reagents were mixed, the SBF was filled to 1 L with DI water. SBF was stored at 37 °C and used within 2 days. The pH was adjusted to 7.4 before use at 37 °C.

Grade 4 and 5 titanium (Ti) particles were suspended in SBF at concentrations of 0.75, 1.5 and 3 mg ml^−1^ in airtight polyethylene containers, which were placed on an orbital shaker rotating at 120 rpm inside a 37 °C incubator. One millilitre of aliquots was taken at day 0, 3 and 10 for the analysis of pH and ionic concentration.

### Cell culture and viability assay

Human gingival fibroblasts (HGFs) (PCS-201-018^TM^, ATCC®, UK) were culture expanded in basal Dulbecco’s modified Eagle’s medium (DMEM) supplemented with 100 unit ml^−1^ penicillin, 100 μg ml^−1^ streptomycin and 10% (v/v) foetal bovine serum (FBS) in standard tissue-culture flasks in a humidified atmosphere containing 5% CO_2_. Upon confluence, cells were subcultured using 1× trypsin-EDTA (500 μg ml^−1^ trypsin with 200 μg ml^−1^ EDTA in Hank’s Balanced Salt Solution).

To evaluate the potential cytotoxic effect of grade 4 and 5 Ti particles on HGFs, two groups of test samples were prepared for cell culture, the particles in media and the dissolution products of the particles. Ti particles were sterilised with 70% ethanol for 1 min before use. Group 1 (dissolution): grade 4 and 5 Ti particles were suspended in DMEM at concentrations of 0.75, 1.5 and 3 mg ml^−1^ in airtight polyethylene containers, which were placed on an orbital shaker rotating at 120 rpm. inside a 37 °C incubator for 72 h. The media were filtered through 0.2 μm PTFE membrane syringe filters following incubation to remove the particles before use in cell culture. Group 2 (particle): Sterilised grade 4 and 5 Ti particles were suspended in DMEM at concentrations of 0.75, 1.5 and 3 mg ml^−1^ and used for cell culture without filtering. Basal DMEM and DMEM containing unprocessed grade 4 and 5 implants were used as controls. One millilitre of aliquots was taken at day 0, 3 and 10 for the analysis of pH and ionic concentration.

A 3-(4,5-dimethylthiazol-2-yl)-2,5-diphenyltetrazolium bromide (MTT) metabolic activity assay was performed to assess cellular metabolic activity of HGFs in response to group 1 and 2 test samples. Briefly, HGFs were seeded at 3000 cells cm^−2^ in 24-well plates and allowed to grow for 24 h in basal DMEM. Basal medium was then replaced with the test samples supplemented with 10% FBS and cultured for a further 3, 7 or 10 days. At each time point, culture medium was replaced with 1 mg ml^−1^ MTT in plain DMEM. MTT solution was removed after 3 h, and dimethyl sulfoxide (DMSO) was added to dissolve the formazan crystals formed by living cells. The luminescence of the resulting solutions was measured at 570 nm in a plate reader.

### Inductively coupled plasma optical emission spectrometer (ICP-OES)

The elemental concentration of titanium, aluminium, iron and vanadium in the following media was determined using ICP-OES (iCaP6300 Duo, Thermo Fisher Scientific, UK) (1) SBF; (2) dissolution products of the particles from immersion in DMEM for 3 days (dissolution), and (3) DMEM sampled during the cell culture studies where cells were cultured with the particles (particle). Mixed standards of titanium, aluminium, iron and vanadium ions were prepared at 0, 2, 5, 20 and 40 ppm for calibration. All samples were run in triplicates, and an acid blank was incubated under the same conditions and used as a control.

### Statistical analysis

Results were presented as mean ± standard deviation (S.D.). Mann–Whitney *U* test (2 groups) or Mann–Whitney *U* test with Bonferroni correction (> 2 groups) was performed using OriginPro 2019. Results were deemed significant if the probability of occurrence by random chance alone was less than 5% (i.e. *p* < 0.05).

## Results

**Fig. 2:**
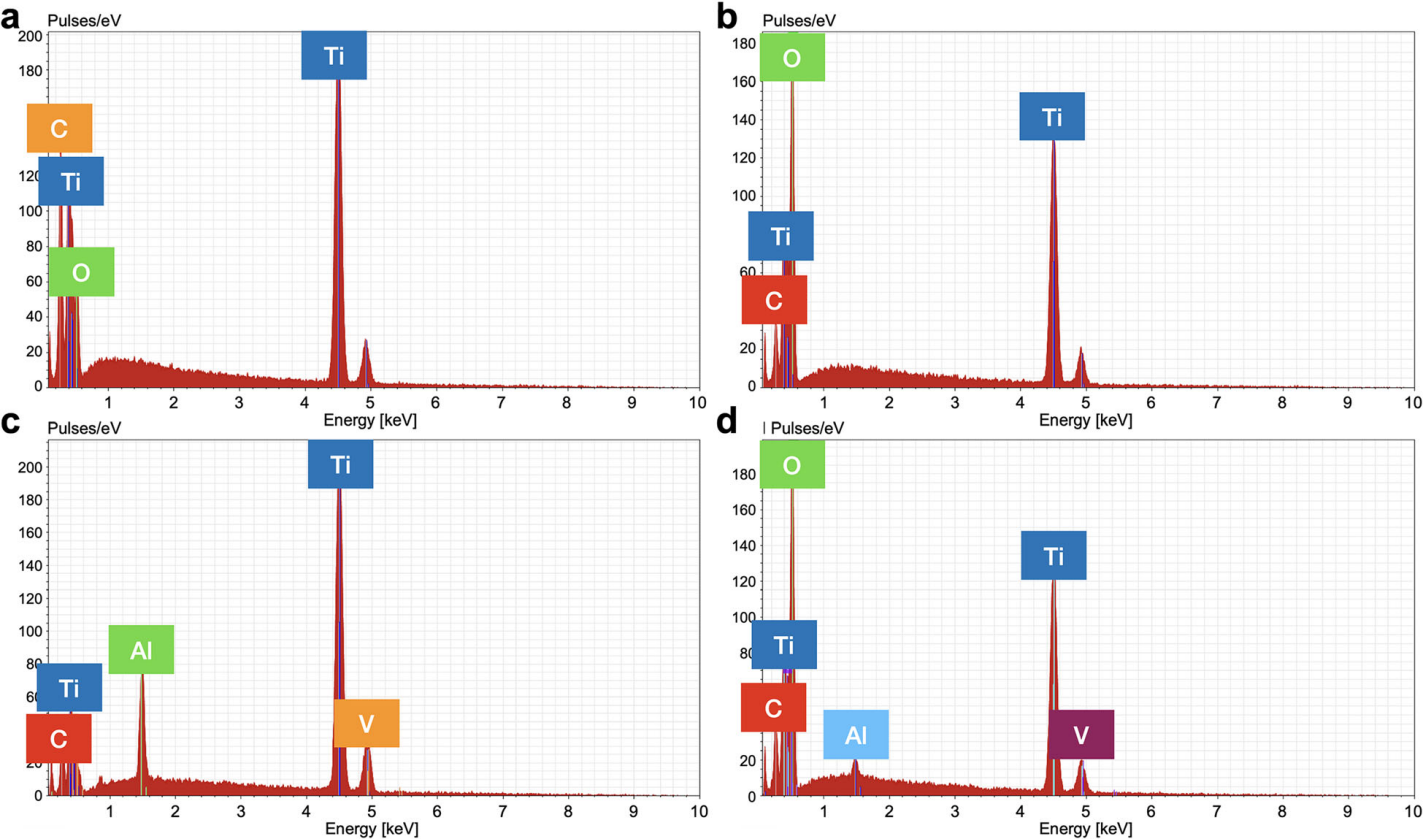
EDX spectra of particles produced by the mock implantoplasty procedure (SEM images in Fig. 1). **a**, **b** Particles from grade 4 commercially pure titanium implant, **a** angular microparticles and **b** small spheres. **c**, **d** particles from grade 5 titanium alloy, **c** angular microparticles and **d** small spheres (grade 5)

Particles released from implants following the mock implantoplasty procedure were collected, and microparticle size of particles produced from the grade 4 (G4) and grade 5 (G5) implants was 77.4 ± 9.1 μm (modal number 66.3 μm) and 48.4 ± 6.4 μm respectively (modal number 43.1 μm). DLS analysis showed nano-sized particles were also present: hydrodynamic diameters were 125.4 ± 10.9 nm (modal number 109.3 nm) and 57.74 ± 2.66 nm (modal number 52.1 nm) respectively. SEM images (Fig. 1) revealed that particles fragmented from both G4 and G5 implants as the result of implantoplasty were irregular in shape and varied significantly in sizes. The elemental composition of the particles was analysed using EDX (Fig. 2). The EDX spectra demonstrated distinctive differences between G4 and G5 particles. In addition to titanium (Ti), both aluminium (Al) and vanadium (V) were detected in G5 particles. Carbon (C) was detected in both samples. According to EDX analysis, the spherical objects amongst implant particles (indicated by arrows in Fig. 1) were titanium oxide.

**Fig. 3:**
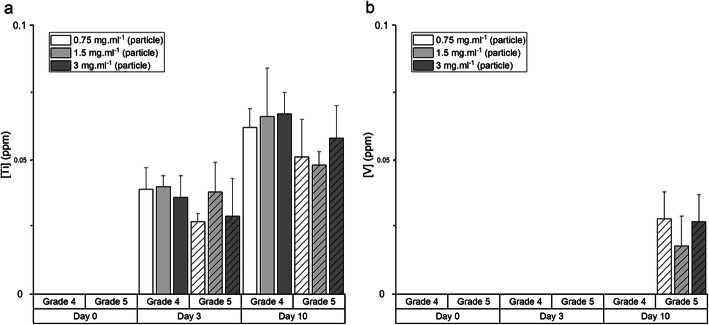
Titanium (Ti) and vanadium (V) release from the particles in simulated body fluid (SBF). Experimental duration was 10 days. Results presented as mean ± standard deviation, *n* = 3

**Fig. 4:**
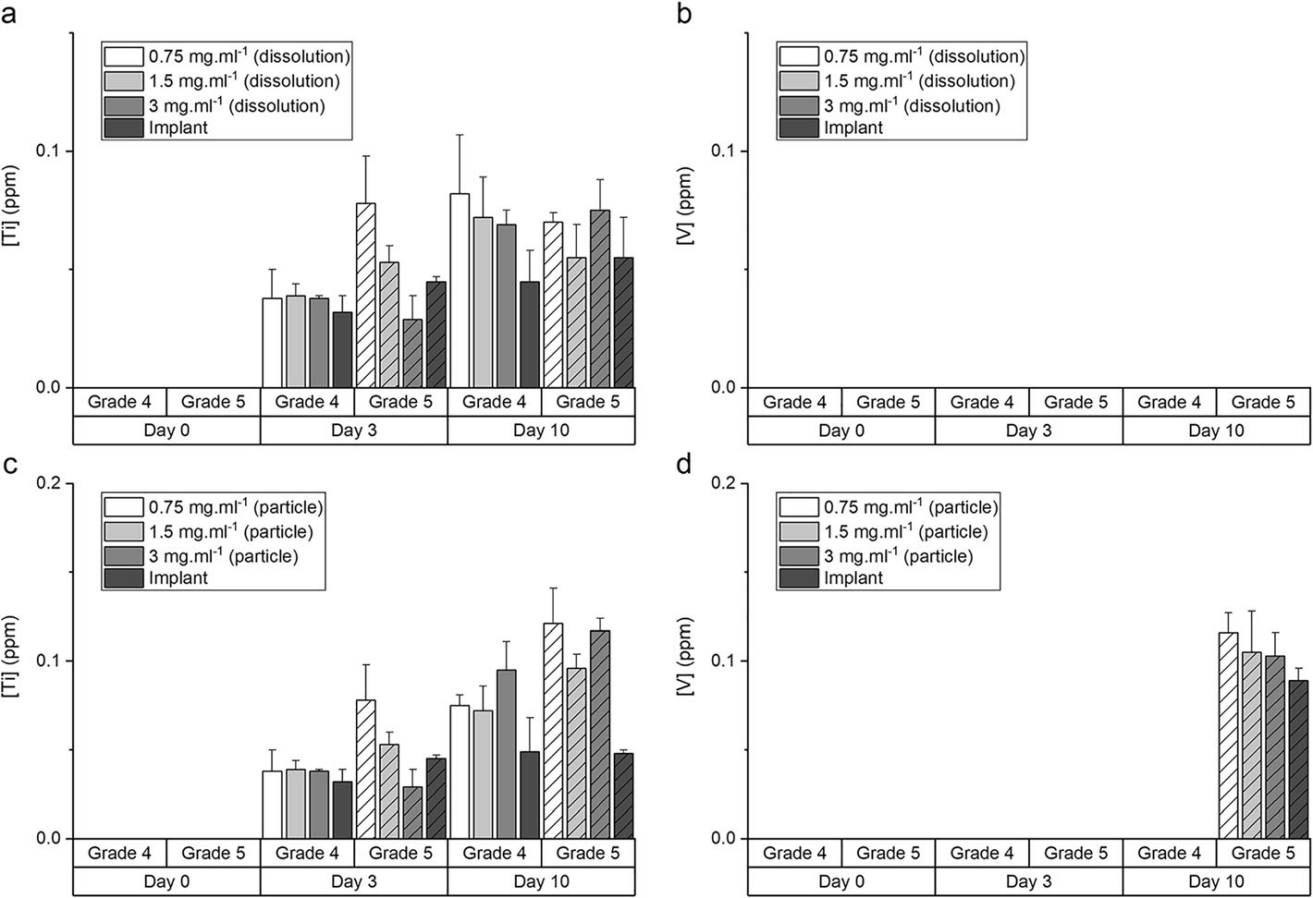
Titanium (Ti) and vanadium (V) content in Dulbecco’s Modified Eagle Medium (DMEM). **a**, **b** Dissolution products (media filtered through 0.2 μm PTFE membrane following initial soaking of the particles for 3 days) and **c**, **d** DMEM sampled during cell culture studies where cells were cultured with the particles over a period of 10 days (particles removed prior to ICP measurement). Results presented as mean ± standard deviation, *n* = 3

Release of ions from the G4 and G5 particles was first investigated in SBF (Fig. 3). Upon immersion, the release of Ti was minimal from both G4 and G5 particles in SBF. Release of V from G5 particles was detected by ICP only after 10 days of immersion. There was no noticeable release of aluminium (Al) and iron (Fe) ions. The pH of SBF remained at 7.4 during the immersion period. Similar results were observed when the particles were immersed in DMEM (Fig. 4), with low release of Ti and no noticeable release of aluminium Al and Fe from both G4 and G5 particles. V, up to 0.116 ± 0.023 ppm, was only detected in medium containing G5 implant particles. Changing the concentration of the particles had little effect. As Al results were negligible, we assumed that no alloy particles passed through the 200-nm filters, but TiO_2_ nanoparticles may have contributed to the Ti values (DLS measurement of blank media control returned value of 0).

**Fig. 5:**
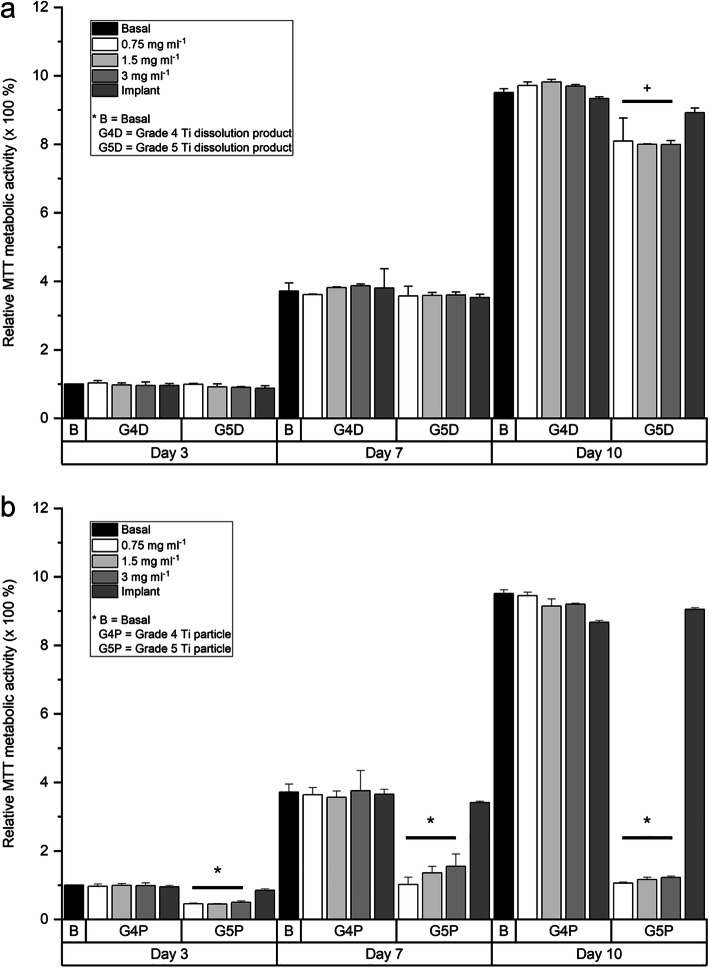
The effect of grade 4 and grade 5 implant particles on human gingival fibroblast viability in vitro. Viability was determined using an MTT metabolic activity assay. Cells were exposed to either **a** dissolution products (ions and nanoparticles) or **b** culture medium containing suspended implant particles throughout the duration of the culture period. Cells were exposed to various concentrations of particles (0.75, 1.5 or 3 mg ml^−1^). Basal medium and basal medium containing unprocessed dental implants were used as controls. All results were normalized against the value of basal medium at day 3. Results presented as mean ± standard deviation, *n* = 3. The asterisk indicates *p* < 0.05, and ^+^ indicates 0.05 < *p* < 0.1 when compared to basal medium control at each time point

The effect of G4 and G5 particles and their dissolution products on human gingival fibroblast viability in vitro was investigated using an MTT metabolic activity assay (Fig. 5). Administration of the dissolution products had no effect on cellular metabolic activity up to day seven, but there appeared to be a reduction in metabolic activity in cells exposed to G5 dissolution products by day 10, though it was not statistically significant. When the cells were exposed to G5 particles, metabolic activity significantly reduced as early as day 3. However, when cells were cultured with the G4 particles, there were no adverse effects. The significantly reduced metabolic activity as a result of exposure to G5 particles was observed at all-time points. Changing the concentration of the particles had little effect.

## Discussion

Unalloyed titanium, often referred to as commercially pure grade 4 titanium (CpTi), usually contains some trace elements of carbon, oxygen, nitrogen and iron (American Society for Testing and Materials international standards). These trace elements improve the mechanical properties of CpTi and are found in higher amounts from grade 1 to 4 CpTi [26]. Many dental implants are made from grade 4 (G4) CpTi, in order to improve its fatigue strength, and companies have also used grade 5 (G5) titanium alloys (Ti-6Al-4 V), which contains metals including vanadium and aluminum.

In the present study, standardised implantoplasty procedure was performed on both G4 (Straumann, model number 021.4512, bone-level implant diameter 4.1 mm, Regular CrossFit®, SLA® 12 mm Roxolid®) and G5 (Biohorizons, model number PBR50105, RBT 5.0 × 12 mm, 5.7 Platform) implants. Implant particles were released from both types of implants (Fig. 1). The smaller particle size generated from G5 implants is likely to be due to the G5 alloy having a higher hardness. Typical G5 alloy has a hardness of 36 (HRC, Rockwell C) compared to 23 (HRC, Rockwell C) of G4 [27, 28]. Some TiO_2_ spheres were detected. This is because when titanium implant surface is exposed to air, titanium oxide (TiO_2_) film is formed on the implant surface. This layer (1.5–10 nm thickness) is formed due to the high affinity of Ti for oxygen [29]. This in turn could contribute to a certain degree of resistance to corrosion of titanium implants [30, 31]. G5 implant particles contain vanadium (Fig. 2), and vanadium ions were released into simulated body fluid and DMEM following 10 days immersion (Fig. 3 and 4).

HGFs exposed to dissolution products of G4 and G5 implant particles did not experience reduction in viability (Fig. 5). This is due to no detectable vanadium release in DMEM by G5 particles during the preparation of the dissolution media, which was 3 days soaking in DMEM (Fig. 4a, b). There was no distinct difference in the amount of titanium ions released from G4 and G5 particles. Direct exposure to G5 implant particles in culture did result in significantly reduced cell viability at all-time points, from 3 to 10 days of culture, while G4 implant particles demonstrated no adverse effect on cell viability (Fig. 5b). The cytotoxic effects of vanadium are well documented [32, 33], so the further relative reduction in cell viability, compared to control and G4 particles, from 10 days was thought to be due to continued release of V during the culture (there was no distinct difference in the amount of titanium ions released from G4 and G5 particles). The delayed release of the V agrees with corrosion studies on Ti-6Al-4 V alloys, which reported time-dependent corrosion [31, 34].

There have been suggestions that sufficient irrigation around the implant and suction of metallic debris during surgical procedures, such as implantoplasty, might reduce the deposition of metallic particles and ions into surrounding tissue [35]. However, there is no scientific evidence that such measures can fully remove implantoplasty debris from the surround environment, and these procedures can also disseminate bacteria into the surrounding tissues [5, 36]. Previous studies indicated that the cytotoxicity of vanadium ion is dose dependent, where concentrations of 23–30 μM (1.17–1.53 μg ml−^1^) significantly reduced cell viability in NIH3T3 fibroblasts [33, 37]. Herein, the V concentration was approximately 0.1 μg ml−^1^ after 10 days of exposure to DMEM, which does explain the significant reduction in viability of HGFs.

Previous studies suggested the viability of calvarial rat osteoblasts in direct contact with G4 CpTi particles (unspecified grade, particle diameter 3.1 ± 3.6 μm) with a concentration higher than 1.5 mg ml−^1^ decreased significantly due to rapid phagocytic process [38]. In the present study, the viability of HGFs appeared to be not affected by G4 CpTi implant particles, even at high concentrations (3 mg ml−^1^). This is due to the lack of vanadium and possibly larger particle size. Here, G5 particles are. Although a range of particle size of G5 was measured in this study, a portion of the particles generated from the mock implatoplasty process in the current study is comparable to that reported by Pioletti et al. [38]. The internalisation of G5 particles, especially sub-micron particles, and the subsequent local release of Vanadium ions inside the cytoplasm could increase the toxicity of the particles. A change in pH of culture medium was not responsible for the reduced viability as the value of pH was found affect by neither G4 nor G5 implant particles.

As discovered by Malvern Mastersizer and DLS, the sizes of G4 and G5 implant particles released as result of implantoplasty are in the range of nano (NPs) and fine particles (FPs) (125.4 ± 10.9 nm–77.4 ± 9.1 μm and 57.74 ± 2.66 nm–48.4 ± 6.4 μm for G4 and G5 implant particles, respectively). NPs and FPs can enter cells via a number of routes such as phagocytosis, endocytosis and macropinocytosis as well as passive diffusion [39]. Exposure to nanomaterials and nanoparticles can potentially result in biological responses at molecular level such as DNA methylation, histone post-translational modifications and noncoding RNAs in mammalian cells [40, 41].

In the field of Orthopaedics, titanium (oxide) wear particles from implants were reported to enter bone-forming cells and stem cells via endocytosis and cause adverse biological response including osteolysis [42–45]. It has also been proposed that titanium particles induce secretion of pro-inflammatory cytokines by fibroblasts, which are involved in the chemotactic migration and recruitment of monocytes/macrophage and subsequently the pathogenesis of aseptic loosening of implants [46]. Therefore, although no short-term effect of G4 particles on HGFs was observed in the present study, further, long-term investigations are also of crucial importance. Further, the effect of implant particles on other cell types within the oral and systemic environments should not be overlooked. Although the health hazards of FPs and NPs are relatively less well established, literature in the fields of toxicology does indicate a glimpse of possible toxicity that should compel clinicians to carefully weigh the possible adverse human health effects. Lastly, “impurities” have been reported in titanium based implant materials, for example, up to 0.5 wt% iron (Fe) in commercial pure titanium (grade 1, 2 and 4) and 0.013 wt% nickel (Ni) (grade 1 and 2) [36, 47, 48]. While these trace metals may not present health risk in a whole implant, ions and particles released as a result of corrosion and/or mechanical intervention such as implantoplasty have been reported to cause adverse allergic reactions in humans [49, 50]. There is no current consensus agreement on the risk of particles released from Ti; however, it would be prudent for clinicians to carefully evaluate the materials used and to consider the potential risks of the individual constituents of any alloy, as indicated in this study.

## Conclusion

In the present study, the release of nano and fine particles from both commercially pure grade 4 titanium and grade 5 Ti-6Al-4 V alloy implants following implantoplasty procedure was reported. Exposure to grade 5 implant particles resulted in significantly reduced cell viability compared to exposure to grade 4 particles. One of the major challenges facing implant dentistry is the lack of information on the possible adverse health effects caused by the exposure to these nano-sized particles. Authors acknowledge that it is inaccurate to directly extrapolate the current findings into human subjects in clinical settings. However, it is probable that the potential toxicity of Ti-6Al-4 V and/or fine implant particles in vivo are also due, at least in part, to the same mechanism presented in the current study. For patient safety, the potential cytotoxicity of Ti-6Al-4 V alloy particles must be considered when used as a material for dental implants. Furthermore, regardless of implant material, the implantoplasty procedure can release nano-sized particles, the full systemic effect of which is not fully understood. Particles have been shown to have local toxic effects; therefore, the authors do not recommend implantoplasty as a safe procedure for the treatment of peri-implantitis.

## Data Availability

The datasets used and/or analysed during the current study are available from rdm-enquiries@imperial.ac.uk on reasonable request.

## Abbreviations

HGF: Human gingival fibroblasts
G5: Grade 5
SEM: Scanning electron microscopy
SBF: Simulated body fluid
V: Vanadium
C: Carbon
DMEM: Dulbeccos’s modified Eagle’s medium
MTT: 3-(4,5-dimethylthiazol-2-yl)-2,5-Diphenyltetrazolium bromide
DMSO: Dimethyl sulfoxide
ICO-OES: Inductively coupled plasma optical emission spectrometer
CpTi: Commercial pure titanium
G4: Grade 4
DLS: Dynamic light scattering
EDX: Energy dispersive x-ray
Al: Aluminium
Ti: Titanium
FBS: Foetal bovine serum
S.D.: Standard deviation
TiO_2_: Titanium oxide
